# Cow’s milk allergy immunoglobulin E-mediated: intake of proteins and amino acids

**DOI:** 10.1590/1806-9282.20220080

**Published:** 2022-08-19

**Authors:** Elaine Cristina de Almeida Kotchetkoff, Raquel Bicudo Mendonça, Talita Lemos Neves Barreto, Renata Magalhães Boaventura, Roseli Oselka Saccardo Sarni

**Affiliations:** 1 Universidade Federal de São Paulo, Pediatrics Department – São Paulo (SP), Brazil.; 2 Universidade Estácio de Sá de São Paulo – São Paulo (SP), Brazil.; 3 Centro Universitário Faculdade de Medicina do ABC – Santo André (SP), Brazil.

**Keywords:** Amino acids, Children, Cow’s milk allergy, Dietary intake, Food hypersensitivity

## Abstract

**OBJECTIVE:**

Children with cow’s milk allergy may be at nutritional risk due to the lower intake of nutrients, such as protein, calcium, and vitamin A, which are present in cow’s milk. The objective was to evaluate children’s diets with Children with cow’s milk allergy compared with healthy controls as well as to compare the intake of proteins and amino acids from the diet followed by Children with cow’s milk allergy who consume special infant formula or plant-based dairy alternatives with Children with cow’s milk allergy who do not consume special infant formula or plant-based dairy alternatives.

**METHODS:**

Through a cross-sectional controlled study, the dietary intake of 57 children (27 with immunoglobulin E-mediated Children with cow’s milk allergy and 30 healthy controls) was evaluated. Using 24-h nutritional recalls, the total energy intake value, macronutrients, and amino acids were calculated.

**RESULTS:**

No statistically significant difference was found between the Children with cow’s milk allergy group and healthy controls for the intake of proteins and amino acids. However, the Children with cow’s milk allergy do not consume special infant formula or plant-based dairy alternatives group had a lower protein (g/kg) and branched-chain amino acid (mg/kg) intake than the Children with cow’s milk allergy consume special infant formula or plant-based dairy alternatives group.

**CONCLUSIONS:**

The Children with cow’s milk allergy group achieved the recommendations for the intake of proteins and amino acids compared to the healthy control group. However, the Children with cow’s milk allergy do not consume special infant formula or plant-based dairy alternatives group had a lower intake of protein (g/kg) and branched-chain amino acid (mg/kg) than the Children with cow’s milk allergy consume special infant formula or plant-based dairy alternatives group.

## INTRODUCTION

The literature well-documented that food allergy (FA) affects more children than adults^1^, and cow’s milk (CM) causes most food allergic reactions in childhood. The treatment for cow’s milk allergy (CMA) is based on excluding CM and dairy products.

The impact of exclusion diets on children’s growth has been emphasized in some studies which found lower height-for-age and weight-for-height than children without exclusion diets^2,3,4,5,6^.

When evaluating the protein intake, it is crucial to go beyond the amount consumed as the composition of amino acids from the diet and protein digestibility are equally important factors^7^.

Given the evidence of greater nutritional risk for children with CMA present in the literature, the lack of studies that assess the protein intake from a quantitative and qualitative point of view, considering the amino acids intake and protein digestibility, the present study was developed. The objective was to compare the dietary intake of proteins and amino acids of children with CMA with healthy controls as well as to compare intake of proteins and amino acids of children with CMA who consume special infant formula or plant-based dairy alternatives (CMA c-SIF/PBDA) with CMA who do not consume special infant formula or plant-based dairy alternatives (CMA dc-SIF/PBDA).

## METHODS

Through a cross-sectional controlled study, 27 patients (aged from 0 to 8 years) with CMA mediated by immunoglobulin E (IgE) were evaluated according to their clinical history and history of sensitivity to CM or oral challenge test for positive CM, who visited the Allergy and Clinical Immunology Outpatient Clinic of the Federal University of São Paulo (UNIFESP) and the Menino Jesus Child Hospital, São Paulo, Brazil. Data were collected between July 2016 and January 2018. The control group consisted of 30 healthy volunteers regularly enrolled in a private school in the city of São Paulo, matched by sex and age.

Children with allergies to food other than milk, who were breastfed, used corticosteroids 3 months before data collection, and had disabsorptive diseases, such as celiac disease, cystic fibrosis, and inflammatory bowel diseases, were excluded from the survey. Those with chronic and acute diseases were excluded during data collection concerning the control group.

The Research Ethics Committee of Unifesp approved the study under protocol 2,621,736, and the Assent Form was applied to all participants and/or guardians before collecting data by a trained nutritionist.

Parents completed a nonconsecutive 3-day dietary recording to evaluate dietary intake, two during the week and one at the weekend^8^. Dietary intake was calculated using Microsoft Excel^®^ with a national database^9^.To calculate the intake of amino acids, the Food Processor^®^ software was used with the United States Department of Agriculture (USDA) database.

To assess the adequacy of dietary protein intake, the mean values obtained were compared to the reference values proposed by the Institute of Medicine (IOM). To measure the adequacy of dietary intake of amino acids, the mean values obtained were compared to the reference values of FAO/WHO^7^.

The Protein Digestibility Corrected Amino Acid Score (PDCAAS) was calculated based on the value of the most limiting essential amino acid chemical score of each protein source. PDCAAS was calculated by multiplying the lowest essential amino acid score by protein digestibility. Protein with PDCAAS ^3^1.0 was of good quality^7^.

To estimate the number of portions of CM and dairy or CM replacements, the portions proposed by Tucunduva et al.^10^ for CM and dairy by age group were used.

To compare the protein sources consumed between the groups, the average protein intake over 3 days was divided into vegetable proteins (all foods of vegetable protein source, except the foods here called CM replacements, namely, plant-based special infant formula and plant-based alternatives), animal proteins (all foods of animal-protein origin, except those here called CM and dairy, namely, dairy-protein-based infant formulas, growing-up milk, *in natura* CM, and all CM dairy products), and other protein sources (an assortment of infant formula, plant-based alternatives, growing-up milk, CM, and dairy products) in g/kg.

### Statistical analysis

Data were entered and consolidated in an Excel spreadsheet (Office Microsoft^®^) and analyzed using the statistical package SPSS 19.0 (IBM^®^). Categorical variables were presented as absolute and percentage values. Continuous variables were analyzed for their normality using the Shapiro-Wilk test. For comparisons between groups, variables with parametric distribution were presented as mean and standard deviation and compared using the independent Student’s t-test. Variables with nonparametric distribution were presented as median (minimum and maximum) and compared using the two-tailed Mann-Whitney U test. The statistical significance level of 5% (p<0.05) was adopted.

## RESULTS

Table 1 describes the general characteristics of 27 children in the CMA group.

**Table 1.** Characteristics of children with cow’s milk allergy (n=27).

		Distribution
Sex (%)	Male	20 (74.1)^a^
Age	Years	4.0±1.9^b^
Ethnicity (%)	Caucasian	15 (55.5)^a^
	Black	6 (22.2)^a^
	Pardo	6 (22.2)^a^
Age group (%)	0–<2 years	4 (14.8)^a^
	2–<6 years	21 (77.8)^a^
	6–10 years	2 (7.4)^a^
Exclusive breastfeeding	Months	4.3 (0.0–7.0)^c^
Total breastfeeding	Months	13.0 (0.0–48.0)^c^
First allergic reaction age	Months	4 (1.0–10.0)^c^
First allergic reaction symptoms	Cutaneous	18 (66.7)^a^
	Systemic	5 (18.5)^a^
	Gastrointestinal	3 (11.1)^a^
	Respiratory	1 (3.7)^a^
CM replacement	SBR	14 (51.8)*
	NR	5 (18.5)^a^
	ISPF	5 (18.5)^a^
	EHF	3 (11.1)^a^
	FAA	1 (3.7)^a^

^a^ n (%): Number (percentage); ^b^Mean±standard deviation; ^c^Median (minimum; maximum). FAA: free amino acid formula; ISPF: Isolated Soy Protein Formula; EHF: extensively hydrolyzed formula; SBR: soy-based replacement; NR: no replacement. *1 child was using these replacements combined with Isolated Soy Protein Formula.

Regarding the CM substitute used by the CMA group, 51.8% of this group used some plant-based replacement. Furthermore, all these substitutes were soy-based (Table 1).

Regarding dietary intake (Table 2), it was observed that children in the CMA group had, compared to the healthy control group, a higher intake of vegetable protein in g/kg [1.43 g/kg (0.62–3.90) vs. 1.14 g/kg (0.47–1.98); p=0.001] and animal protein in g/kg [1.66 g/kg (0.68–3.57) vs. 1.46 g/kg (0.10–3.96); p=0.035]. When analyzing the consumption of proteins from CM and dairy products or CM replacements, it was observed that the CMA group, compared to the healthy control group, had a lower intake of proteins from CM replacements in g/kg (0.67±0.42 g/kg vs. 1.32±0.69 g/kg; p≤0.001). Also, the CMA dc-SIF/PBDA group had lower protein intake in g/kg (2.95±0.63 g/kg vs. 4.45±1.54 g/kg; p=0.044) than the CMA c-SIF/PBDA group.

**Table 2.** Comparison between children with cow’s milk allergy and nonallergic controls of nutritional status, dietary intake, dietary intake of amino acid, and quality of protein consumed.

		Cow’s milk allergy group (n=27)	Group control (n=30)	p
Anthropometry				
Age	Years	4.0±1.9^d^	4.0±1.8	0.958^a^
Body mass index	Z-score	-0.03 (-2.3–2.7)^5^	0.17 (-2.5–2.3)	0.867^b^
Body mass index classification (%)	Eutrophic	19 (70.1)	21 (70.0)	0.441^c^
	Thinness	1 (3.7)	4 (13.3)	
	Overweight	6 (22.2)	4 (13.3)	
	Obesity	1 (3.7)	1 (3.7)	
Height/age	Z-score	-0.24 (-2.1–0.5)	0.19 (-2.4–1.9)	0.004^b^
Stature classification (%)	Normal height	25 (92.6)	28 (93.3)	
	Short stature	2 (7.4)	2 (6.7)	0.653^c^
Dietary intake				
Total energy	Kcal/day	1615.3±427.3	1568.3±393.2	0.667^a^
Carbohydrate	%TCV	50.8 (39,2–66.7)	53.5 (42.2–85.9)	0.379^b^
Lipid	%TCV	33.1±4.8^d^	31.9±4.1	0.312^a^
Protein	%TCV	15.2 (10.2–27.3)	15.6 (12.6–20.8)	0.554^b^
Total protein	g/kg	3.8 (2.1–7.3)	3.9 (2,0–6.4)	0.701^b^
Vegetable protein*	g/kg	1.43 (0.62–3.90)	1.14 (0.47–1.98)	0.001^b^
Animal protein**	g/kg	1.66 (0.68–3.57)	1.46 (0.10–3.96)	0.035^b^
CM protein, dairy, or replacements	g/kg	0.67±0.42	1.32±0.69	<0.001^a^
Dietary intake per meal				
Breakfast and snacks				
Energy	Kcal	814.3±253.4	890.9±258.1	0.264^a^
Carbohydrate	%	30.2±7.3	32.4±6.0	0.224^a^
Lipid	%	14.4±3.8	17.9±4.6	0.002^a^
Protein	%	5.0±1.3	6.8±2.2	<0.001^a^
Protein	g/kg	1.30 (0.46–2.40)	1.64 (0,42–3.88)	0.059^b^
Lunch and dinner				
Energy	Kcal	822.1±240.8	678.2±239.8	0.028^a^
Carbohydrate	%	20.8±4.7	19.9±6.2	0.545^a^
Lipid	%	18.9±4.4	13.9±3.9	<0.001^a^
Protein	%	10.7±3.3	9.1±2.7	0.053^a^
Protein	g/kg	2.54 (1.39–5.70)	2.23 (0,68–4.09)	0.057^b^
number of CM portions or replacements		1.9±0.7	3.4±1.5	<0.001^a^
Amino acids				
Isoleucine	mg/kg	113.5 (32.2–237.7)	91.1 (24.7–284.4)	0.213^b^
Leucine	mg/kg	194.5 (61.9–379.2)	164.5 (45.3–485.2)	0.284^b^
Valine	mg/kg	122.8 (38.4–240.5)	107.6 (32.7–303.8)	0.330^b^
Aromatic amino acids	mg/kg	83.6±32.0	80.7±41.8	0.772^a^
Sulfur-containing amino acids	mg/kg	187.7 (70.6–387.3)	169.8 (53.7–508.4)	0.701^b^
Histidine	mg/kg	71.1±30.4	70.6±38.8	0.952^a^
Lysine	mg/kg	165.2 (48.8–322.7)	137.6 (33.9–389.3)	0.666^b^
Threonine	mg/kg	88.8 (29.2–173.5)	72.5 (21.6–202.3)	0.462^b^
Tryptophan	mg/kg	22.8 (8.5–52.3)	21.3 (6.8–57.3)	0.620^b^
BCAA	mg/kg	143.8 (44.2–285.8)	120.1 (34.2–357.8)	0.277^b^
Essential amino acids	mg	13970.0 (5283.3–25576.6)	11470.0 (3343.3–27953.3)	0.672^b^
Total amino acids	mg	35530.0 (13040.0–63630.0)	29936.6 (10083.3–71406.6)	0.632^b^
Protein quality				
PDCAAS		0.60±0.19	0.64±0.25	0.523^a^

^a^Student’s t-test; ^b^Mann-Whitney test; ^c^χ^2^; ^d^Mean (standard deviation); Median (minimum and maximum). *Except proteins from special infant formula and plant-based alternatives. **Except proteins from CM and dairy products. TCV: Total Caloric Value; CM: cow’s milk; BCAA: branched-chain amino acid; PDCAAS: protein digestibility corrected amino acid score. Bold indicates statistically significant values.

When comparing the intake grouped by meals, it was observed that, at breakfast and snacks, the CMA group, compared to the control group, had a lower intake of protein in g/kg (5.0±1.3% vs. 6.8±2.2%; p≤0.001).

The CMA group, compared to the healthy control group, intake fewer portions of CM replacements (1.9±0.7) vs. CM and dairy group (3.4±1.5) p≤0.001.

No statistically significant difference was found regarding the intake of the amino acids in mg/kg, when the CMA group was compared with the healthy control group (Table 2). However, when the CMA dc-SIF/PBDA group (n=5) was compared with the CMA c-SIF/PBDA group (n=22) (Table 3), there was a statistically significant difference. There was lower intake of all branched-chain amino acids (BCAA) as follows: isoleucine (75.1±34.9 mg/kg vs. 129.9±48.4 mg/kg; p=0.025), leucine (126.3±57.9 mg/kg vs. 220.4±79.8 mg/kg; p=0.020), and valine (85.3±37.8 mg/kg vs. 140.3±50.3 mg/kg; p=0.031). There was no statistically significant difference in anthropometric indexes between the CMA dc-SIF/PBDA and the CM c-SIF/PBDA groups.

**Table 3.** Evaluation of dietary intake of amino acids and proteins, protein quality, and anthropometric indexes between children with cow’s milk allergy who do not consume special infant formula or plant-based dairy alternatives (n=5) and cow’s milk allergy who consume special infant formula or plant-based dairy alternatives (n=22)

		CMA dc-SIF/PBDA (n=5)	CMA c-SIF/PBDA (n=22)	p
Amino acids (mg/kg)				
Histidine		48.6±23.7	76.3±29.8	0.064^a^
Isoleucine		75.1±34.9	129.9±48.4	0.025^a^
Leucine		126.3±57.9	220.4±79.8	0.020^a^
Valine		85.3±37.8	140.3±50.3	0,031^a^
Aromatic amino acids		64.4±27.6	87.9±31.9	0.141^a^
Sulfur-containing amino acids		146.9±65.2	217;3±83.5	0.091^a^
Lysine		115.4±63.7	180.5±69.4	0.067^a^
Threonine		65.5±31.5	100;4±36.7	0.061^a^
Tryptophan		18.7±8.6	27.7±11.8	0.119^a^
BCAA		95.6±43.4	163.5±59.4	0.024^a^
Protein/quality				
Protein (g/kg)		2.95±0.63^c^	4.45±1.54	0.044^a^
PDCAAS		0.61±0.32^c^	0.59±0.16	0.953^a^
Anthropometry				
Body mass index	Z-score	0.32±1.47	0.08±1.15	0.687^a^
Height-for-age	Z-score	-0.14 (-2.08–0.47)^d^	-0.27 (-2.05–0.46)	0.755^b^

^a^ Student’s-t-test; ^b^Mann-Whitney test; ^c^Mean (standard deviation); ^d^Median (minimum and maximum). CMA: cow’s milk allergy; dc-SIF/PBDA: do not consume special infant formula or plant-based dairy alternatives; c-SIF/PBDA: consume special infant formula or plant-based dairy alternatives. Bold indicates statistically significant values.

## DISCUSSION

In this present study, no differences were found in the dietary intake of proteins and amino acids between the CMA and the healthy control groups, nor in the quality of protein consumed. However, the CMA dc-SIF/PBDA group had a lower protein (g/kg) and BCAA (mg/kg) intake than the CMA c-SIF/PBDA group.

Results similar to those observed in this study were previously reported by Rowicka et al.^11^ in CMA group. The authors highlighted that the protein intake by children with CMA exceeded three times the recommendation. In contrast, other authors have mentioned lower protein intake by CMA children than those without dietary exclusion^12,13,14^.

Regarding the analysis of amino acids from the diet of children with CMA, according to the best of our knowledge, there are no studies published to date.

Intake of amino acids is closely related to children’s growth, as the amino acid recommendations for this age group should be considered^7^. In a study of 313 children from Malawi, who aged between 12 and 59 months (62% of them with low ZH), serum levels of amino acids were measured. The authors observed lower levels of all essential amino acids in the low ZH group than those with adequate growth^15^.

A cross-sectional study with 5034 healthy Canadian children aged between 24 and 72 months verified the association between the intake of non-cow milk beverages and shorter stature in childhood. The authors observed that each cup of non-cow milk beverage consumed daily was associated with a 0.4 cm reduction in the children’s height compared to those who consumed CM^16^. In our study, some CM replacements used by the CMA group may explain this group’s lower stature, which in some cases was inadequate.

When evaluating protein intake, we must consider the origin of the protein, as it is generally agreed that animal-derived proteins play an important role in children’s growth. It is known that the amino acids present in CM, especially the BCAA ones, influence the production of somatomedin C or IgF1, with a positive impact on growth^17^. When we assessed the dietary sources of proteins consumed by the groups studied, we observed that the CMA group had a higher intake of vegetable protein (from cereals and other foods, except from CM replacements) and animal protein (from meat and eggs) compared to the healthy control group. Opposite results were found by Maslin et al.^18^, which compared the dietary intake between children with a CM elimination diet and children without elimination. It was verified that the group on the CM elimination diet had less animal protein intake than the group without the elimination diet.

In the Netherlands, a cohort evaluated the impact of the intake of different protein sources in 3564, 1-year-old children on BMI and height at 9 years. It concluded that the early high intake of animal protein was associated with higher BMI and height at 9 years old than those with predominant intake of vegetable protein^19^.

In our study, the CMA group had lower protein at breakfast and snacks, which may be explained by the smallest number of portions of CM replacements intake consumed by these individuals. Similar results were found by Vassilopoulou et al.^20^, who assessed the effects of FA on the eating habits of children aged 6–11 years. Children with FA had lower protein intake at breakfast and snacks than controls. In the CM replacement group, the absence of these meals was balanced by the intake of juice and dried fruits. Similarly, in our study, we observed that five children were not using any CM replacements in the CMA group. However, the recommendation in g/kg of protein consumed was achieved due to the higher intake of animal and vegetable proteins present in other meals.

When individuals in the CMA dc-SIF/PBDA group were compared with those in the CMA c-SIF/PBDA group, it was observed that children with CMA dc-SIF/PBDA had a lower intake of protein in g/kg and of BCAA in (mg/kg of weight body), with no differences in anthropometric indexes. This difference between the CMA groups must be interpreted carefully, considering that both achieved the amino acid recommendations in mg/g of protein by intake of another protein source. However, it opens a possibility for future studies for evaluating the serum levels of these amino acids in children with CMA on an elimination diet.

The present study was a pioneer in describing the intake of amino acids in children with CMA compared with healthy controls. Still, it has the following limitations: cross-sectional design, limitations of the PDCAAS method, absence of a national database with amino acids present in foods, and sample size.

We conclude that the CMA group, under nutritional intervention, did not differ from the healthy control group in terms of intake of proteins and amino acids. However, the CMA dc-SIF/PBDA group had a lower intake of protein and BCAA than the CMA c-SIF/PBDA group.
